# B’More healthy communities for kids: design of a multi-level intervention for obesity prevention for low-income African American children

**DOI:** 10.1186/1471-2458-14-942

**Published:** 2014-09-11

**Authors:** Joel Gittelsohn, Elizabeth Anderson Steeves, Yeeli Mui, Anna Y Kharmats, Laura C Hopkins, Donna Dennis

**Affiliations:** Johns Hopkins Global Obesity Prevention Center, Johns Hopkins Bloomberg School of Public Health, 615 North Wolfe St, Baltimore, MD 21205-2179 USA

**Keywords:** Obesity, Children, Urban, Multi-level interventions, Policy, Study design

## Abstract

**Background:**

Childhood obesity rates in the U.S. have reached epidemic proportions, and an urgent need remains to identify evidence-based strategies for prevention and treatment. Multi-level, multi-component interventions are needed due to the multi-factorial nature of obesity, and its proven links to both the social and built environment. However, there are huge gaps in the literature related to doing these kinds of interventions among low-income, urban, minority groups.

**Methods:**

The B’More Healthy Communities for Kids (BHCK) intervention is a multi-level, multi-component intervention, targeting low-income African American youth ages 10–14 and their families in Baltimore, Maryland. This intervention prevents childhood obesity by working at multiple levels of the food and social environments to increase access to, demand for, and consumption of healthier foods. BHCK works to create systems-level change by partnering with city policy-makers, multiple levels of the food environment (wholesalers, corner stores, carryout restaurants), and the social environment (peers and families). In addition, extensive evaluation will be conducted at each level of the intervention to assess intervention effectiveness via both process and impact measures.

**Discussion:**

This project is novel in multiple ways, including: the inclusion of stakeholders at multiple levels (policy, institutional, and at multiple levels of the food system); that it uses novel computational modeling methodologies to engage policy makers and guide informed decisions of intervention effectiveness; it emphasizes both the built environment (intervening with food sources) and the social environment (intervening with families and peers). The design of the intervention and the evaluation plan of the BHCK project are documented here.

**Trial registration:**

NCT02181010 (July 2, 2014).

## Background

Currently, 35.0% of adults in the United States are obese and 33.6% are overweight [1]. While US obesity rates have leveled off in recent years [1], the high prevalence of obesity remains a severe threat to the health of Americans. Simply stated, obesity is caused by an imbalance in energy intake and expenditure; however, there are multiple, complex factors that influence this equation. The rise in obesity in the US has occurred too rapidly to be primarily related to our biology [2], which has led scientists and practitioners to examine changes in the food environment, policies, and production system as potential drivers [2, 3]. Over the past 40 years the U.S. food system and food environment has evolved to provide an abundant supply of inexpensive, highly palatable, high energy foods that are accessible, convenient, and heavily marketed [3]. In this context, high obesity rates may be viewed as a natural response to the environment [3].

The food environment may be defined as the types of food sources that are accessible to an individual (such as supermarkets, fast food restaurants, convenience stores, school meal programs, etc.) and what consumers are exposed to in those environments (availability of healthy and unhealthy foods, prices, promotions/marketing, etc.) [2, 4]. A review of the literature by Larson and colleagues [5] found that increased access to supermarkets was related to improvements in diet quality, fat intake, and fruit and vegetable consumption in studies of both adults and adolescents. Increased access to grocery stores is generally linked to reduced levels of obesity, whereas increased access to convenience stores, corner stores, and fast food outlets is linked to increased obesity [5–9]. Additionally, low-income and minority neighborhoods have disproportionately lower levels of access to healthier food sources (i.e. supermarkets) and increased access to less healthy food sources (i.e. fast food, convenience stores, corner stores) [5, 10–12], which may contribute to the disparities seen in obesity rates among groups.

Like many cities in the US, healthy foods and food source availability is limited in low-income areas in Baltimore [13–16]. These areas have few supermarkets, but do have many small food sources including corner stores, carry-out restaurants, and fast food restaurants [13–15]. Corner stores in Baltimore have limited space and stock primarily high fat and high added sugar items [17]. Baltimore carry-outs primarily serve high fat and high sugar foods [18]. In 2007, 32.7% of Baltimore City adult residents were overweight and 35.0% were obese [19]. These disparities can be attributed, in part, to the poor community food environment.

It is clear that multi-level, multi-component interventions are needed to address the obesity epidemic, and that this work needs to take place in low-resource settings. In the area of childhood obesity prevention, the majority of work has taken place in schools – with limited impact [20]. In recent years, a number of trials have combined school-based approaches with complementary approaches in the community [21]. Shape Up Somerville was one such successful approach. Using community participatory approaches, Shape up Somerville was designed to prevent undesirable weight gain by intervening within the before-, during-, and after-school environments of an elementary school child. Program development involved the engagement of various community members, including school food service providers, before- and after-school programs, restaurants, parents, children, and others. As a result, the multi-component program led to a decrease in BMI percentile among children [22]. Despite this success, fewer than half of the combined school-community intervention trials have successfully reduced obesity in children. Some limitations of this previous work include: lack of attention to policy, which is needed for long-term sustainability and stakeholder buy-in; no systematic exploration of the potential for different intervention strategies to work alone and in combination; lack of emphasis on the social environment as part of intervention strategies, weaknesses in the delivery of the intervention, such that intensity and exposure is limited, and a near total lack of work on low income urban populations, where the risk for obesity and associated negative outcomes is the greatest. New approaches are needed that will work at multiple levels, combine multiple intervention venues and strategies – and that address the significant gaps presented above.

**Figure 1:**
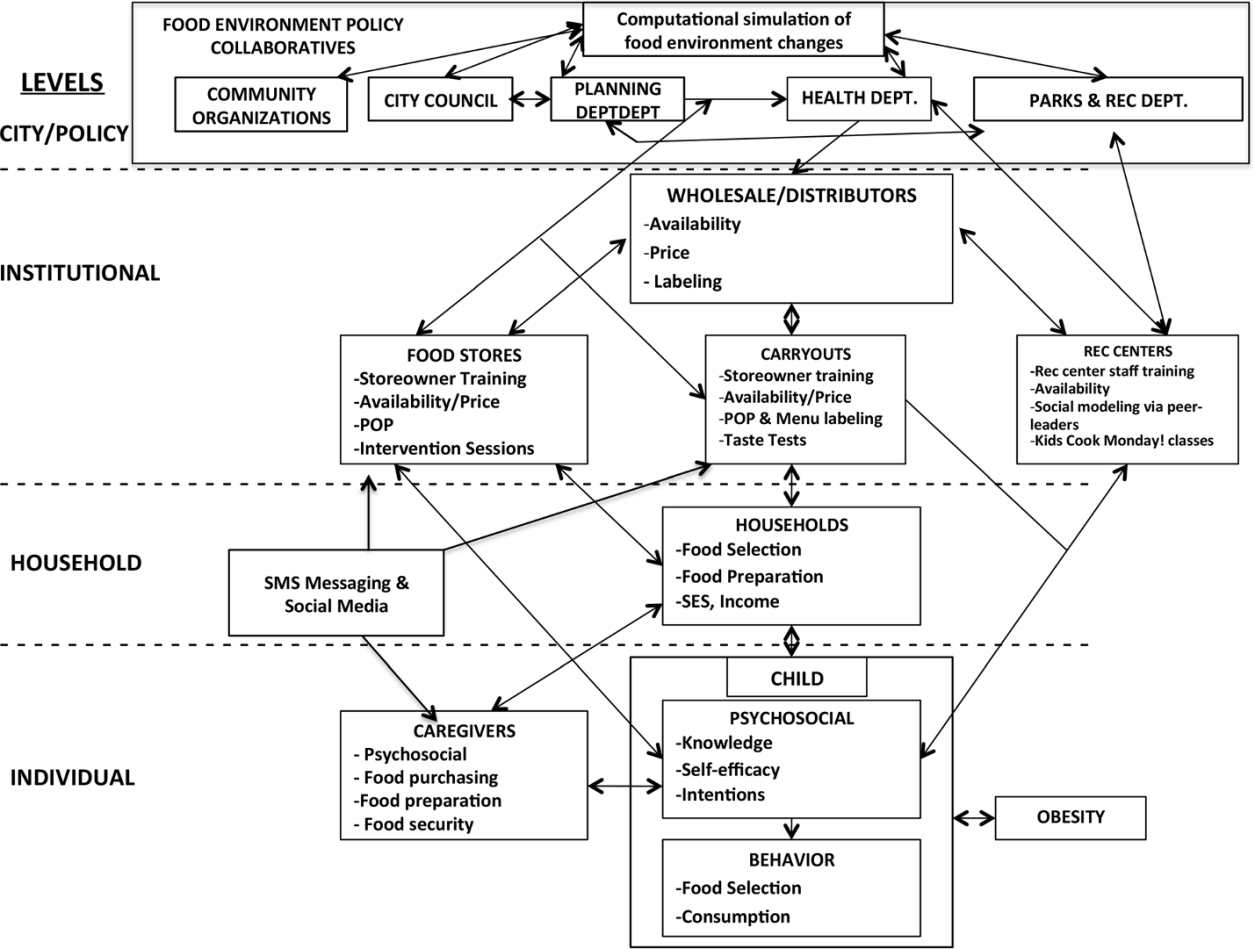
**B’More healthy communities for kids conceptual framework.**

The B’More Healthy Communities for Kids (BHCK) trial is a multi-level child obesity prevention intervention, supported as part of an U54 grant to the Johns Hopkins Bloomberg School of Public Health to fund the Johns Hopkins Global Obesity Prevention Center. The BHCK intervention is guided by social cognitive theory (SCT), social ecology, and systems theory [23–26]. SCT and social ecology conceptualize the individual as nested within broad social and environmental networks and structures that impinge on their perceptions, outlooks, and behaviors (Figure 1). Psychosocial factors (e.g. knowledge, intentions and self-efficacy), social-environmental factors (e.g. behavioral observations), and physical-environmental factors (e.g. food availability, price) interact at various levels to shape health outcomes [26–32]. According to systems theory, this dynamic and complex system operates as one whole interacting functional unit, in which information and influences flow bi-directionally from one level to another. Policy, institutional, and behavioral strategies have the potential to reach multiple levels of the food environment. The BHCK trial seeks to develop and test a series of intervention strategies that will function at multiple interacting levels, and will be implemented in collaboration with city officials, local wholesaler(s), and retail food stores/carryouts. These strategies will improve the healthy food supply chain for low-income communities. Institutional level intervention components (e.g., wholesalers, retailers, recreation centers, and consumers) will promote healthy dietary behaviors in order to influence the household (e.g., food purchasing and preparation) and individual food-related psychosocial factors and behaviors, which will ultimately impact risk of obesity. Emphasis will be placed on institutional and behavioral strategies, (e.g., retailer discounts, point of purchase promotions, and health education), which will elicit change at each level.

## Methods/design

The BHCK trial uses a group randomized study design, where 30 low-income, geographic zones surrounding recreation centers have been selected to serve as either intervention or comparison areas. All areas were required to be predominantly low-income and African American. Half of these zones have been randomized to intervention (Healthy Eating Zones (HEZs)), while the other half have been set to comparison (delayed intervention), in two waves. The center point (recreation center) of all intervention and comparison zones must exceed one mile in distance from each other, to reduce the potential for contamination.

The research was approved by the Johns Hopkins Bloomberg School of Public Health Institutional Review Board. Written informed consent was obtained from all adult participants for themselves, and for children. Written assent was obtained from all children who were part of the evaluation sample.

### Formative research

Extensive formative research was conducted to aid in program planning and materials development. Methods included in-depth interviews and focus groups with youth and their adult caregivers, ground truthing of food sources, and environmental scans of food availability. In-depth interviews with youth (ages 10–14) and their adult caregivers were conducted to identify key areas for intervention, along selection of the most appropriate communication channels and messaging materials. Ground truthing techniques were used to identify and map food sources in each intervention and comparison neighborhood with over 300 unique food sources documented.

Selection of appropriate and acceptable foods to be promote during the intervention is critical to the success of this project. To select the food items that we will promote in the intervention, study staff observed inventory at local wholesalers to identify availability of healthy and affordable beverages, snacks, and grocery items. Four child focus groups were conducted at Baltimore City recreation centers. Children were involved in the selection of foods for promotion, and in communication material pre-testing, to gauge which images, characters, slogans, and messaging resonated.

Three additional focus groups were conducted with adult caregivers to discuss media preferences for information dissemination, information needs, and to ascertain interest in cooking classes. During the final two groups, parents were asked to provide feedback on draft text-messages, and to discuss how frequently they would like to receive the text-messages.

### Participants and recruitment

The target group in the BHCK multi-level intervention are low-income African American adolescents, ages 10–14, and their adult caregivers. Recruitment will occur at recreation centers and neighborhoods sites within each zone. Interested parents/guardians and youth are screened for eligibility prior to enrollment. Once a sample of 75 youth are screened as eligible in each of the HEZs, a sampling frame will be created for that HEZ. Among those recruited and screened, 24 dyads (comprised of a child and adult pair) will be randomly selected in each neighborhood. If a randomly selected dyad was unable to complete the interview, then the next eligible dyad will be chosen from the recruitment list.

### Intervention

The BHCK intervention will involve multiple components at many levels (Figure 1): policy, wholesalers, recreation centers, corner stores/carryouts and family/parents. Each component of the BHCK program will reinforce several other components – either by improving access or increasing demand.

#### Policy level approaches

The policy-level intervention component will bridge the gap between research and policy by engaging with and informing key Baltimore City stakeholders. The policy work will strive to develop collaborations to sustain BHCK program elements, and to contribute to policy to improve the food environment. A working group of nearly forty members was established in July 2013 with representation from the Baltimore City Health Department, City Council, Department of Planning, school food services, non-profit organizations, and academia. Quarterly meetings and cross-sector communications with collaborators ensure engagement.

A key role of JHU will be the provision and development of the evidence base to support decision-making and policy development by these entities. Unique to this component will be the use of computational modeling to simulate the potential impact of programs and policies for reducing children’s obesity risk. Using agent-based modeling [33], we have developed a virtual representation how low income Baltimore children behave in their food environment, including dietary and physical activity choices. Key institutions within this model (corner stores, carryouts, recreation centers) can be impacted by different intervention strategies. The model will serve three major purposes as: 1) a novel tool to better understand the dynamic nature of children and their food environment; 2) a low-cost approach to predict the impact of obesity prevention programs and policies before investing in implementation; and 3) a highly visual, interactive, and intuitive platform to facilitate the exchange of ideas with policymakers. The model will be presented at regular meetings to solicit feedback and promote dialogue about programs and policies of interest to stakeholders. Activities of the workgroup, including the number of attendees per meeting, the number of divisions represented at each meeting, suggested updates to the model by stakeholders, requests to simulate program or policy impact by stakeholders, etc. will be tracked to measure progress of this intervention component (Table 1).

**Table 1:** **Selected process evaluation measures and minimum standards per intervention level**

Intervention level	Intervention component	Minimum standards for delivery
Policy	# of attendees/meeting	>25
	# of different sectors represented/meeting	>6
	# ABM sub-groups formed/year	>2
Wholesaler	# of food items that meets the nutrition guidelines per phase	≥3
	# wholesalers that provide discount to BHCK intervention stores	2
	% of shelf labels correctly placed	≥75%
Recreation Center/Peer Leader	# of planned intervention sessions delivered by youth-leaders	≥75%
	# of kid interactions per session at the recreation center	≥12
Corner Store/Carryout	# of NEW promoted foods stocked per phase	≥4
	# of kid interactions during interactive session/store/phase	≥20
	# healthier options on menu (designated by green leaf)/phase	≥4
	# reduced price healthier options on menu/phase	≥4
Family/SMS messaging, social media	% families receiving invitation to join SMS program	90%
	% that join	40%
	# SMS text messages sent to participants/week	2
	% text messages received	80%
	% families that participate in one of BHCK’s social media websites	50%
	# posts/week on 1+ social media websites	1
	# goal-setting messages/week	1

#### Wholesaler engagement and pricing strategies

Our work with wholesalers will aim to increase access to healthier foods by small retail and prepared food sources in the city, by increasing the stocking and sales of affordable healthy food options at local wholesalers that supply corner stores and carryouts. BHCK will collaborate with two local wholesale distributors and a national club store. These organizations have agreed to stock at least one type/brand of the promoted food items for each sub-phase of the program and to allow us to post the BHCK logo sign on the shelves above or adjacent to the promoted products. BHCK staff will visit these locations once per month to assess the stocking of promoted foods and to ensure that logo signs remain posted correctly.

Small store retailers often pay higher prices for (low-demand) healthy foods than larger retailers due to their lower inventory. Participating wholesalers have agreed to provide discounts on select BHCK promoted food items to participating small food sources. We will also provide storeowners with gift cards from these businesses to use to purchase an initial stock of the promoted items. Expected benefits to participating wholesalers include increased sales, potential new customers, and public recognition for their support of the project.

#### Recreation center activities and work with peer leaders

The BHCK study has used a participatory process with community partners and young people from intervention neighborhoods to develop the youth-leader intervention including the youth-leader training materials, a curriculum that will be delivered to recreation center youth, and messaging and promotional media (videos, posters). For youth in the 10–14 year old age range, youth-leaders are seen as a reliable, relatable and credible source of information [34]. We will train a cohort of 16 youth-leaders (local college students, ages 18–22 years) to deliver a nutrition intervention to the target population of youth in the recreation centers in each intervention neighborhood.

Youth-leaders who successfully complete the training program will go on to deliver the curriculum to youth in the recreation centers. The recreation center sessions will involve a brief instructional period (5–15 minutes of information giving) followed by interactive games, activities, taste tests, and cooking classes to reinforce promotional messages. These sessions will be delivered by the youth-leaders bi-weekly with the support of project staff. The topics, activities, and taste tests that occur in the recreation centers will mirror the topics and foods/beverages promoted in the store intervention.

Additionally, the youth-leaders’ roles will extend beyond the recreation centers and will cut across the intervention components, as they will also be involved in delivering the intervention components in stores, via promotional materials, and on social media. The youth-leaders will partner with BHCK staff to deliver interactive sessions in the corner stores, they will serve as models or “spokespeople” for the program by having their images featured on promotional materials (posters, Facebook posts, tweets), and will generate and promote (by liking and sharing) social media content for the intervention.

#### Changing food access in corner stores/carryouts

At the food sources level, BHCK will aim to increase access to, and demand for healthier food options. A minimum of three food sources (at least two corner stores and one carryout) will be recruited from each zone, located within a ½ mile radius of the recreation center. The intervention components at the store level are based on previous corner store [35–39] and carryout [40–42] trials completed in Baltimore City, with additional innovative pieces.

At the small food source level, BHCK will begin with a series of storeowner trainings which aim to improve their knowledge of healthier food options and self-efficacy to be able to stock, prepare and sell their foods. These trainings will be developed based on formative research [40–43], and will address the following topics: *1) Introduction to B’More Healthy Communities for Kids; 2) Customer Service Strategies for Success; 3) How to Keep Your Food Safe, Fresh, and Healthy; 4) Business Strategies for Success: How to Stock Healthier Foods; 5) How to Get WIC in Your Store;* and *6) Improving Your Store Environment*. Following completion of the trainings, store owners may choose structural incentives to aid in the stocking of healthier food items, i.e. a banana holder, grill, or small produce refrigerator.

After completion of the training phase, the food promotion phases will begin, which include Smart Beverages, Smart Snacks, and Smarter Cooking Methods. During each phase, corner storeowners will be asked to stock at least four new healthy food options. At the carryouts, store owners will be asked to make the default beverage option a healthier option (i.e. water with meal instead of soda), provide healthier side dishes, engage in healthier cooking methods, such as grilling, and create a healthy combo meal on their menu. In order to create demand for these foods and improve customers’ knowledge about the foods, a messaging campaign, which was tested in focus groups with adults and children, will be implemented through posters, improved menu boards, shelf talkers, labels, and other signage. Interactive sessions, such as taste tests or blind tasting challenges, will occur in each store at least every other week. If stores successfully stock new, healthy food options and allow the BHCK team to promote the foods with their stores throughout the phases, the BHCK team will progressively deem each store a Bronze, Silver, Gold, or Platinum Certified Healthy Store.

#### Family-level work and text messaging

Adult caregivers will receive bi-directional text-messages (Textit, Inc. [44]), and have the option to select from one of two frequencies of text-message delivery: twice a week or three to five times a week. The campaign content will be modified over the course of the intervention and will be tailored to each neighborhood. The first message each week will encourage completion of an attainable and specific goal. For example, “Does ur child have a sweet tooth? Try offering them granola bars or fruit as an alternative to candy 1 time this week”. The subsequent text-messages will offer support to help parents reach the goal, by highlighting discounts offered on promoted products at local stores, or BHCK related activities.

Social media websites such as Facebook, Twitter, and Instagram will mirror the content of the text-messages. Images will be added to the websites and interaction between parents will be encouraged. Participants will be asked to share whether they were able to achieve weekly goals and advise other parents. The websites will also be used to link caregivers to outside resources available through other organizations, (i.e. farmers markets that accept Supplemental Nutrition Program Assistance benefits, cooking classes offered for adults through the American Heart Association).

### Standards for intervention delivery

Intervention implementation at each level will be monitored through ongoing process evaluation, with the intent to assure that set standards are being met (Table 1). These standards are based on our review of the literature, and on our previous experience with wholesaler, corner stores, carryout and peer-led interventions.

### Comparison group (delayed Intervention)

The comparison areas will receive an abbreviated version of the BHCK intervention following completion of the post-intervention evaluations.

### Measurements

The BHCK trial will be evaluated at each level (Table 2). Process evaluation measures will assess reach, dose delivered and fidelity of intervention implementation (Table 1).

**Table 2:** **Impact evaluation components for the BHCK trial per intervention level**

Intervention Level	Impact/outcome measures
Policy	% of action items achieved/year
	# of health-related issues put on policymaker’s agenda/year
	# of health-related issues introduced by policymaker/year
Wholesaler	% change in sales of promoted foods based on collected sales data from wholesalers
	% change in sales of promoted foods to BHCK corner stores and carryouts
Recreation Center	Changes in recreation center policies regarding the food environment
	Changes in the recreation center food environment (e.g., after-school snack program, concession stand foods)
Corner Store/Carryout	# of posters, shelf labels, shelf talkers, etc. seen by BHCK participants (exposure)
	# promoted foods purchased/consumed by BHCK participants
	# of giveaways received by BHCK participants
	# units of promoted foods sold
Adult/household (SMS)	Household food purchasing (healthy and unhealthy foods)
	Healthiness of common methods of food preparation
	Change in psychosocial factors (knowledge, self-efficacy, intentions)
	Change in weight, BMI
Children and Youth Leaders	Frequency of purchase of healthy and unhealthy foods
	Healthiness of food preparation methods
	Change in dietary patterns (e.g., total calories, total fat, FV servings, HEI scores, etc.)
	Change in psychosocial factors (knowledge, self-efficacy, intentions, outcome expectations)
	Change in BMI percentile

A sample of adult caregiver-child dyads (n = 24 dyads per zone, 720 at baseline) will be surveyed pre- and post-intervention to assess impact. The first section of the adult instrument contains questions pertaining to household size and composition, and use of community recreation centers by children. Specific food sources visited for purchasing/getting foods and amount of money spent at each source is recalled for the last 30 days. Frequency of purchase of 40+ non-prepared foods in the last 30 days is recalled (fruits, vegetables, chips, soda, chicken, etc.). The next section asks how many times a meal was prepared for the household in the last 30 days, and inquires about the top three most common preparation methods of seven types of foods (chicken, fish, potatoes, etc.). A series of self-efficacy questions assess confidence in performing healthier behaviors along a 4 point ordinal scale. Eight questions about behavioral intentions about food are asked (i.e., “The next time you fried an egg, what would you use to fry it? a) Cooking Spray; b) Oil; c) Butter, Margarine, Shortening, or Lard.” Ten food-related knowledge questions are asked. The next section asks a series of questions about health beliefs and attitudes using a Likert scale, i.e. “Health foods are tasteless. Do you Strongly Disagree, Disagree, Undecided, Agree, or Strongly Agree?” Adult respondents are questioned about their food assistance participation and are asked to provide basic socioeconomic information (education level, income category). The last section of the survey contains the 18 question USDA food security questionnaire [45].

The child interview consists of two instruments – the Block Kids 2004 Food Frequency Questionnaire (FFQ) and a Child Impact Questionnaire (CIQ). The Block Kids 2004 FFQ instrument is a validated FFQ (Nutrition Quest, Berkeley, CA) that asks about frequency and portion of consumption of 77 food items as is based on NHANES 1998–2002 data [46]. The CIQ consists of 79 questions pertaining to demographics, food purchasing, food preparation, intentions about food, outcome expectancies, self-efficacy, food knowledge, social support, and breakfast consumption. Demographics including age, birthdate, sex, race, and contact information are obtained. Specific food sources visited, number of times patronized, and who the child most frequently shopped within the last seven days is recalled. Frequency and location of purchase of 65+ non- prepared and prepared food items is recalled for the last seven days (beverages, fruits and vegetables, groceries, fast food, snacks, etc.). The next section of the questionnaire assesses how often a member of the household prepared food for the child and how often the child prepared food for themselves in the last seven days. If a child prepared food for themselves, information on types of food prepared and methods of preparation utilized are collected. Twelve questions about behavioral intentions about food are asked (i.e. “If you wanted a snack, which would you choose? a) Potato chips; b) Pretzels; c) Yogurt). Eleven questions on both short-term and long-term outcome expectancies related to consumption of healthy and unhealthy foods are asked (i.e. I would be healthier if I ate French fries three times a week instead of eating French fries seven days a week. Is this a) True; b) Mostly true; c) Mostly false; or d) False). A series of twelve self-efficacy questions assess confidence in performing healthier behaviors along a 4 point ordinal scale. Fourteen food-related knowledge questions are asked. The survey contains two social support scales. The first scale consists of seven questions that inquire whether the child has someone in their life that would support healthier food and physical activity habits, who that person is, and whether they are older, younger, or around the same age as the child. The second social support scale consists of fourteen questions and asks how often the child’s parents and peers exhibit certain behaviors that support healthy and unhealthy eating using a Likert scale. The final section assesses frequency of breakfast consumption and includes a 24-hour recall of breakfast consumed the previous day. Anthropometric data (height and weight) are collected from both the caregiver and the child using a Seca 213 Portable Measuring Rod stadiometer and a Tantia BF697W Duo Scale.

At the store-level, sales data will be collected in two ways. Before, during and after the intervention, a BHCK staff member will ask store owners approximately monthly to recall sales of selected food items (promoted and non-promoted foods) in the last 7 days. Additionally, some of the wholesalers with which we partner will provide the BHCK team with specific sales data from accounts (stores) throughout the intervention. Wholesalers have agreed to provide sales data on promoted foods for BHCK participating corner stores and carryouts.

### Sample size and statistical methods

We used baseline data from our previous trial, Baltimore Healthy Stores, regarding adult food purchasing to calculate sample size and study power to address the second hypothesis. Based upon our sample size calculations, we will need to have a sample size of 720 adult caretaker-child dyads (this is equivalent to 24 dyads from each HEZ) for the intervention assessments. Assuming a 15-20% drop-out after two years, this will leave us with a minimum of 600 adult caretaker-child dyad respondents post-intervention. We will be able to detect a 4–6 point change in our frequency of healthy food purchasing score, reflecting one healthy food purchased once a week.

For children participants in the dyads, sample size and power for program impact on children’s diet was calculated using national data on low-income urban AA youth diet. Within each selected household, we will randomly sample one child in the 10–14 year age range. Assuming 600 child respondents post-intervention, we will be able to detect a 320–450 difference in caloric intake, a difference of 12–15 g of fat intake and a difference of 1–1.5 percentage points in percent of energy from fat.

## Discussion

To our knowledge, BHCK will be one of few multi-level, multi-component obesity prevention intervention trials working with urban, low-income, minority youth. First, BHCK will be a unique trial that seeks to integrate stakeholders at multiple critical levels: policy; food supply (wholesalers); retail (corner stores, carryouts); community (recreation centers); and individual (children and caregivers). Second, policymakers and other key stakeholders will be engaged with the research in a novel manner using computational modeling. Our ability to simulate the potential impact of programs and policies within a virtual food environment will provide an approachable and low-cost means to collaboratively explore ways to improve the food environment. Third, multiple components of the food system will be targeted. Not only do storeowners receive business and nutrition training, but the program intervenes at the wholesale level as well. Fourth, there will be an emphasis on social aspects of the environment, operating at the individual level -- including text messaging with caregivers and delivery of nutrition interventions through incorporation of youth-leaders to allow for enhanced social modeling of desired eating behaviors. This will be one of the first nutritionally related text messaging campaigns that will target low-income participants, and incorporate information regarding participants’ local food environment. Lastly, detailed process and impact evaluations will occur at all intervention levels.

Researchers, public health officials, and policy makers will have a significant interest in the results of the BHCK intervention. Several leading organizations and scientists have cited that multi-level, multi-component interventions are required to address the obesity epidemic in the US, yet few of these large intervention trials have been successfully completed. Trials such as BHCK are needed to support this claim.

One powerful advantage of BHCK intervention, will be that it will serve as a model of how to engage with city policymakers to improve the food environment. Because of its regular and prolonged engagement with policy makers, the BHCK intervention will have the potential to create long-term impact, and to be sustainable through institutionalization of intervention components with the largest impact into our partners in the Baltimore City Health Department and city government.
